# A Pilot Study (SWOG S0429) of Weekly Cetuximab and Chest Radiotherapy for Poor-Risk Stage III Non-Small Cell Lung Cancer

**DOI:** 10.3389/fonc.2013.00219

**Published:** 2013-08-28

**Authors:** Yuhchyau Chen, James Moon, Kishan J. Pandya, Derick H. M. Lau, Karen Kelly, Fred R. Hirsch, Laurie E. Gaspar, Mary Redman, David R. Gandara

**Affiliations:** ^1^Department of Radiation Oncology, University of Rochester, Rochester, NY, USA; ^2^SWOG Statistical Center, Seattle, WA, USA; ^3^Hematology Oncology, University of Rochester, Rochester, NY, USA; ^4^Hematology Oncology, University of California at Davis, Sacramento, CA, USA; ^5^Department of Medicine and Pathology, University of Colorado, Denver, CO, USA; ^6^Department of Radiation Oncology, University of Colorado, Denver, CO, USA

**Keywords:** cetuximab, stage III non-small cell lung cancer, EGFR, performance status, radiosensitization

## Abstract

**Purpose:** Stage III non-small cell lung cancer (NSCLC) patients with poor performance status (PS) or co-morbidities are often not candidates for standard chemoradiotherapy (chemoRT) due to poor tolerance to treatments. A pilot study for poor-risk stage III NSCLC patients was conducted combining cetuximab, a chimeric monoclonal antibody targeting epidermal growth factor receptor (EGFR), with chest radiation (RT).

**Methods:** Stage III NSCLC patients with Zubrod PS 2, or Zubrod PS 0–1 with poor pulmonary function and co-morbidities prohibiting chemoRT were eligible. A loading dose of cetuximab (400 mg/m^2^) was delivered week 1, followed by weekly cetuximab (250 mg/m^2^)/RT to 64.8 Gy in 1.8 Gy daily fractions, and maintenance weekly cetuximab (250 mg/m^2^) for 2 years or until disease progression. H-score for EGFR protein expression was conducted in available tumors.

**Results:** Twenty-four patients were enrolled. Twenty-two were assessed for outcome and toxicity. Median survival was 14 months and median progression-free survival was 8 months. The response rate was 47% and disease control rate was 74%. Toxicity assessment revealed 22.7% overall ≥Grade 3 non-hematologic toxicities. Grade 3 esophagitis was observed in one patient (5%). The skin reactions were mostly Grade 1 or 2 except two of 22 (9%) had Grade 3 acne and one of 22 (5%) had Grade 3 radiation skin burn. Grade 3–4 hypomagnesemia was seen in four (18%) patients. One patient (5%) had elevated cardiac troponin and pulmonary emboli. H-score did not reveal prognostic significance. An initially planned second cohort of the study did not commence due to slow accrual, which would have added weekly docetaxel to cetuximab/RT after completion of the first cohort of patients.

**Conclusion:** Concurrent weekly cetuximab/chest RT followed by maintenance cetuximab for poor-risk stage III NSCLC was well tolerated. Further studies with larger sample sizes will be useful to establish the optimal therapeutic ratio of this regimen.

## Introduction

Multiple randomized phase 3 clinical studies have demonstrated the superiority of combination chemoradiation (chemoRT) to radiotherapy (RT) alone for inoperable stage III non-small cell lung cancers (NSCLC) (1–8). These clinical trials generally have selected patients with good performance status (PS) and no major medical co-morbidities or weight loss. Consequently, combination thoracic radiation (RT) and platinum-based chemotherapy has become the standard treatment approach for this patient population.

However, in everyday oncologic practice, stage III NSCLC patients with poor PS and/or co-morbidities are often not candidates for standard combination chemoRT due to poor tolerance, increased toxicities, and uncertainty of a survival benefit. Thus, the treatment of this fragile yet common subset of stage III inoperable NSCLC has been a clinical challenge. Recognizing this unmet need, The Southwest Oncology Group (SWOG) has conducted a series of clinical trials with modified chemoRT regimens with the intent to identify a tolerable and effective regimen for poor-risk stage III NSCLC patients. Two pilot studies by SWOG were conducted. S9429 evaluated a regimen of RT with concurrent etoposide and the substitution of carboplatin for cisplatin, and S9712 evaluated low-dose weekly paclitaxel and carboplatin with concurrent RT followed by consolidation paclitaxel. S9429 yielded a median survival of 13 months, while S9712 yielded a median survival of 10.3 months. The lower survival time in S9712 was attributed to a 7% treatment-related death rate (9, 10).

With proven efficacy and mild toxicity observed with the epidermal growth factor receptor (EGFR) pathway targeting agents (monoclonal antibodies and tyrosine kinase inhibitors) for the treatment of advanced NSCLC, these agents seem ideal to explore as alternatives to cytotoxic chemotherapeutic agents in the treatment of stage III NSCLC patients with poor-risk features. Cetuximab, a chimerized antibody to EGFR, is attractive because of its proven radiosensitizing properties in head and neck cancer (11). In addition, cetuximab in combination with chemotherapy has demonstrated a modest survival gain compared with chemotherapy alone in a phase 3 trial for chemotherapy-naive patients with EGFR-expressing advanced stage NSCLC (median survival 11.3 vs. 10.1 months, *p* = 0.044) (12). In comparison with cytotoxic chemotherapy, the side effects of cetuximab are relatively mild, usually limited to rash, fatigue, and low magnesium levels.

Given the known benefit of adding systemic therapy to RT in this disease setting, evaluation of a radiosensitizing agent that is devoid of the traditional toxicities of chemotherapy is an attractive approach in poor-risk stage III NSCLC patients. The primary objectives of our study were to assess the feasibility and toxicity of weekly cetuximab and concurrent chest RT. The secondary objectives were to evaluate the response rate as well as overall (OS) and progression-free survival (PFS). If this regimen were to prove feasible and well tolerated, the plan was to add weekly low-dose docetaxel (20 mg/m^2^) to the regimen in a second cohort of patients. We also conducted preliminary EGFR IHC receptor analyses to gain information on the relationship between EGFR expression and survival/PFS in this study cohort with the limited sample size.

## Materials and Methods

This was a National Cancer Institute (NCI) cooperative group trial administered through SWOG. The treatment protocol (clinicaltrials.gov identifier: NCT00288054) was approved by institutional review boards at each participant site. All patients enrolled in the study provided oral and written informed consent.

### Patient eligibility

Adult patients with newly diagnosed and pathologically confirmed inoperable stage IIIA or IIIB NSCLC according to AJCC TNM staging 6th edition (13), defined as poor-risk for chemoRT based on SWOG criteria, were eligible. These included patients with a Zubrod PS 2 had to have FEV1 > 800 ml and corrected DLCO ≥8 ml/mmHg/min; and patients with Zubrod PS 0–1 had to have pulmonary function (FEV 1 < 2 l) and co-morbidities prohibiting chemoRT. All patients were required to have adequate hepatic and hematologic labs, and a calculated creatinine clearance of ≥20 ml/min. Exclusion criteria included patients with malignant pleural effusion (wet IIIB) or any distant metastasis; patients with prior chemotherapy, surgery, or radiotherapy for this diagnosis; patients with adenocarcinoma-*in situ* (bronchioloalveolar carcinoma), stage IIIB tumor involving the superior sulcus (Pancoast) tumors (due to a competing SWOG protocol S0220 for Pancoast tumors); and patients with medical illnesses including but not limited to active infection, unstable congestive heart failure, active angina, unstable cardiac arrhythmias, and peptic ulcer disease uncontrolled by appropriate treatments.

### Study design

The design was a dose escalation study with two cohorts. The initial 27 patients enrolled were to receive a regimen of cetuximab and concurrent daily chest RT followed by maintenance cetuximab. If the regimen of concurrent cetuximab and chest RT was well tolerated, the study would then move forward to the second cohort with concurrent cetuximab, chest RT, weekly low-dose docetaxel, and maintenance cetuximab.

### Pretreatment evaluations

Pretreatment evaluation included a complete medical history and physical examination including PS, weight, CT of chest and abdomen, PET scan, laboratory analysis (CBC with differentiation, electrolyte and blood chemistry, liver enzymes, magnesium, and LDH), pulmonary function tests, brain scan, ECG if indicated, and a bone scan if clinically indicated.

### Cetuximab treatment

A loading dose of cetuximab at 400 mg/m^2^ over a 2-h infusion was given during the first week. Chest radiation started the second week with concurrent weekly cetuximab at 250 mg/m^2^ over a 1-h IV infusion. All patients were premedicated with diphenhydramine 50 mg IV prior to the first dose. Premedication was recommended prior to subsequent doses. The dose of diphenhydramine could be reduced at the treating physician’s discretion. Maintenance weekly cetuximab at 250 mg/m^2^ continued until disease progression or for 2 years after completion of concurrent chest RT.

### Cetuximab dose modifications

Dose modifications for future cetuximab infusions were instituted in case of severe acneiform rash (Grade 3). No dose modification was required for Grade 1 or 2 rash. Treatment with topical and/or oral antibiotics was considered at the discretion of the treating physician. Topical corticosteroids were not recommended. For Grade 2 rash that was unacceptable to the patient for symptomatic reasons, cetuximab was held until resolution to ≤Grade 1 for up to 4 weeks. For Grade 3 skin reaction of the first occurrence, cetuximab treatment was delayed for 1–2 weeks. If the toxicity resolved to ≤Grade 2, treatment may be resumed without a change in dose level. If the toxicity did not resolve, to ≤Grade 1 after 4 weeks, protocol treatment was discontinued. For any second occurrences of a Grade 3 skin reaction, cetuximab treatment was delayed for 1–2 weeks. If the toxicity resolved to ≤Grade 2, treatment was re-initiated at 200 mg/m^2^. If the toxicity did not resolve to ≤Grade 1 after 4 weeks, protocol treatment was discontinued. For the third occurrence of a Grade 3 skin reaction, cetuximab therapy was delayed for 1–2 weeks. If the toxicity resolved to ≤Grade 2, treatment was re-initiated at 150 mg/m^2^. If the toxicity did not resolve to ≤Grade 1 after 4 weeks, protocol treatment was discontinued. If there was a fourth occurrence of a Grade 3 skin reaction, protocol treatment was discontinued.

### Chest radiation

Radiation began on week 2 (week 1 was for the loading dose of cetuximab) and continued through 7.5 weeks. All treatments were based on CT simulation with corrections for tissue inhomogeneity. Gross tumor volume (GTV) was defined as all gross disease including the primary tumor and clinically involved lymph nodes (≥1.5 cm by CT scan, PET positive, or physical exam, or biopsy positive). The clinical tumor volume was defined as GTV with 1.5 cm margins. The planning treatment volume (PTV) was the GTV with 2-cm margins (CTV = GTV + 1.5 cm margins, and PTV = CTV + 0.5 cm margins). The PTV could be treated with any combination of coplanar or non-coplanar three-dimensional (3D) conformal fields shaped to deliver the specified dose while restricting the dose to the normal tissues. The treatment plan used for each patient was based on an analysis of the volumetric dose including the dose volume histogram (DVH) analyses of the PTV and critical normal structures. Standard normal tissue constraints were applied. In cases with extensive atelectasis and/or pneumonia where tumor margins were obscure, field boundaries were left to the judgment of the treating radiation oncologist. RT prescription was PTV to 64.8 Gy in 36 fractions given 5 days a week, 1.8 Gy/day. The dose was prescribed to the isocenter. The dose within the PTV could not vary by more than ±10% of the stated target dose. Prophylactic mediastinal, contralateral hilar, or supraclavicular lymph node radiation was not allowed. Intensity modulated radiation therapy (IMRT) was not allowed. V20 (percent lung volume receiving at least 20 Gy radiation dose) on the radiation treatment plan needed to be ≤30%.

### Follow-up evaluations

Toxicity, PS, body weight, blood counts, and electrolytes were evaluated weekly during the concomitant weekly cetuximab radiation period. Post-radiation evaluation included medical history and physical examination including PS, body weight, lab test, and toxicity evaluation at weeks 10, 14, 18, and 22. After week 22, the monitoring continued once every 3 months with the additional inclusion of CT of chest and abdomen, PET scan if indicated, and brain scan if indicated.

### Tumor response evaluation

Objective response was recorded at each evaluation with the Response Evaluation Criteria in Solid Tumors (RECIST) criteria: (a) Complete Response (CR): complete disappearance of all measurable and non-measurable disease. No new lesions. No disease-related symptoms. (b) Partial Response (PR): applied only to patients with at least one measurable lesion and ≥30% decrease from baseline of the sum of longest diameters of all target measurable lesions. No unequivocal progression of non-measurable disease. No new lesions. All target measurable lesions must be assessed using the same techniques as baseline. (c) Stable Disease: not qualifying for CR, PR, or progression. All target measurable lesions must be assessed using the same techniques as baseline. (d) Progression: one or more of the following must occur: 20% increase in the sum of longest diameters of target measurable lesions over smallest sum observed (over baseline if no decrease during therapy) using the same techniques as baseline (14).

### EGFR protein expression (IHC with Dako pharmDx)

One slide per patient was stained with Dako pharmDx EGFR antibody. One H&E slide for each patient was received for pathologic review as well. The methodology for EGFR IHC analysis has been previously reported (12, 15, 16). Specimen evaluation was performed by a certified pathologist. The preferable number of viable tumor cells in order to qualify for IHC assessment was 50. However, when there were less than 50 cells but more than 20 they were evaluated and a notation was made in the comments section. Tumors were evaluated by two different methods. In the first method, only the membrane staining, whether or not it was completely circumferential, was considered and the percent of total tumor cells within each staining intensity category [0 (no staining), 1+ (weak), 2+ (moderate), 3+ (strong)] was reported (15, 16). In the second method, the cytoplasm was evaluated and the percent of total tumor cells within each staining intensity category [0 (no staining), 1+ (weak), 2+ (moderate), 3+ (strong)] was reported (15, 16). A hybrid-(H) score was generated based on the fraction of staining cells in each intensity category. The H-score was calculated by completing the formula (% cells of 0 intensity × 0) + (% cells of 1+ intensity × 1) + (% cells of 2+ intensity × 2) + (% cells of 3+ intensity × 3). This produces a final H-score with a range from 0 to 300.

### Statistical methods

The objective of this study was to assess the feasibility and toxicity experience for poor-risk stage III NSCLC patients treated with the study regimen. The primary endpoint was defined as the rate of Grade 3 or greater esophagitis or pneumonitis within 4 months after discontinuation of radiation therapy.

The design was a dose escalation study with two cohorts. The initial 27 patients enrolled were to receive a regimen of cetuximab and concurrent RT with no docetaxel, and with maintenance cetuximab for 2 years or until disease progression. If 10 or fewer patients experienced either Grade 3 or greater esophagitis or pneumonitis within 4 months after discontinuation of radiation therapy, a subsequent cohort of 27 patients would be enrolled to receive a regimen of cetuximab, concurrent RT, plus docetaxel (20 mg/m^2^) followed by maintenance cetuximab as described. For each cohort of 27 patients, the toxicity of the regimen would be considered unacceptable if 11 or more patients experienced either Grade 3 or greater esophagitis or pneumonitis within 4 months after discontinuation of radiation therapy. This design was sufficient to distinguish between the null hypothesis that the dose is unsafe (≥55% of patients experiencing Grade 3 or greater esophagitis or pneumonitis toxicity) vs. the alternative of a safe dose (<30% with Grade 3 or greater esophagitis or pneumonitis toxicity) with 84% power, using a one-sided test based on the binomial distribution with a significance level of 5%.

Adverse events were assessed according to the CTCAE (NCI Common Terminology Criteria for Adverse Events) Version 3.0. Only events that were reported as possibly, probably, or definitely related to protocol treatment were included in this analysis.

Progression-free survival and OS were estimated using the Kaplan–Meier method (17). Confidence intervals for the median PFS and median OS were constructed using the Brookmeyer and Crowley methods (18).

An exploratory analysis was performed to investigate the relationship between EGFR protein expression, as measured by H-score, and clinical outcomes. The association with PFS and OS was evaluated using a two-sided log-rank statistic.

## Results

The study was open to accrual from April 1, 2006 to May 15, 2009, a duration of 37 months. The study was closed early due to poor accrual. Twenty-four patients were enrolled in the first cohort and received cetuximab + RT with no docetaxel. Two patients were considered ineligible due to having a baseline FEV 1 > 2 l. Baseline patient characteristics are shown in Table 1. Twenty patients (91%) received the planned radiation dose. Six patients (27%) had cetuximab dose reductions during concurrent RT. Eighteen patients went on to receive maintenance therapy with cetuximab. The median number of cetuximab cycles during maintenance was 5.5 (range: 1–23 cycles).

**Table 1:** **Patient characteristics (*N* = 22)**.

	*N*	Percentage
**AGE**		
Median (range)	72.9 (51.3–84.5)	–
**GENDER**		
Male	11	50
Female	11	50
**RACE**		
White	20	91
Black	1	4.5
Asian	1	4.5
**PERFORMANCE STATUS**		
0	2	9
1	15	68
2	5	23
**SMOKING HISTORY**		
Current	9	41
Former	12	54.5
Never	1	4.5
**TUMOR STAGE**		
IIIA	12	55
IIIB	10	45
**WEIGHT LOSS**		
<5%	13	59
5 ≤ 10%	5	23
10–20%	3	14
>20%	0	0
Not reported	1	5
**FEV1**		
≥2L	19	86
≥1–<2L	2	9
Not reported	1	5
Albumin: median (range)	4.0 (2.7–4.9)	–

### Safety analysis

All 22 eligible patients were assessed for adverse events (AEs). Toxicity assessment revealed 22.7% overall ≥Grade 3 non-hematologic toxicity. One patient had Grade 3 treatment-related esophagitis, and no patients had ≥Grade 3 pneumonitis. Therefore, the estimated rate of Grade 3 or greater pneumonitis/esophagitis was 5% (95% confidence interval: 0–23%). Table 2 summarizes other treatment-related AEs of interest. AEs potentially related to cetuximab included the following: two of 22 (9%) had Grade 3 acne, one of 22 (5%) had Grade 3 skin burn. There was a Grade 3 (5%) hypomagnesemia and 2 (9%) Grade 4 hypomagnesemia. There was no Grade 3 rash. There was one patient with Grade 4 AE with elevated cardiac troponin in a patient who had pulmonary embolism. Most other AEs were Grade 1 and/or 2. There were no treatment-related deaths among the above-mentioned three patients (14%) who had experienced treatment-related Grade 4 adverse events.

**Table 2:** **Adverse events (CTCAE Version 3.0)**.

	Grade 1 *N* (%)	Grade2 *N* (%)	Grade 3 *N* (%)	Grade 4 *N* (%)	Grade 5 *N* (%)
Pneumonitis	1 (5)	4 (18)	0	0	0
Esophagitis	4 (18)	3 (14)	1 (5)	0	0
Cardiac troponin I	0	0	0	1 (5)	0
Thrombosis/embolism	0	0	0	1 (5)	0
Hypomagnesemia	3 (14)	0	1 (5)	2 (9)	0
Dry eye	1 (5)	0	0	0	0
Nail changes	0	2 (9)	0	0	0
Allergic reaction	0	2 (9)	1 (5)	0	0
Fatigue	8 (36)	5 (23)	1 (5)	0	0
Weight loss	2 (9)	2 (9)	0	0	0
**SKIN REACTIONS**					
Acne	7 (32)	6 (27)	2 (9)	0	0
Burn	0	0	1 (5)	0	0
Dry skin	3 (14)	0	0	0	0
Pruritus/itching	3 (14)	1 (5)	0	0	0
Rash/RT dermatitis	3 (14)	4 (18)	0	0	0
**GASTROINTESTINAL**					
Diarrhea	4 (18)	2 (9)	1 (5)	0	0
Dysphagia	4 (18)	3 (14)	0	0	0
Odynophagia	2 (9)	0	0	0	0
**HEMATOLOGIC**					
Hemoglobin	4 (18)	4 (18)	0	0	0
Lymphopenia	0	1 (5)	4 (18)	0	0
Neutrophils	0	1 (5)	1 (5)	0	0
Platelets	2 (9)	0	0	0	0
**RESPIRATORY SYMPTOMS**					
Cough	3 (14)	1 (5)	1 (5)	0	0
Dyspnea	4 (18)	1 (5)	3 (14)	0	0
Hypoxia	0	0	1 (5)	0	0

### Tumor response

Nineteen patients had measurable disease at baseline and were included in the analysis of response. Nine patients had either a confirmed (7) or unconfirmed (2) PR for a response rate of 47% (95% confidence interval: 24–71%), with a median duration of 6 months. Five additional patients had a best response of stable disease for a disease control rate of 74% (95% confidence interval: 41–91%). The other five patients included two patients with increasing disease, and three patients who could not have their exact response determined due to inadequate disease assessments but were included in the denominator as non-responders. This study utilized the RECIST 1.0 criteria and response was assessed only in those patients with measurable disease at baseline.

### Survival

**Figure 1:**
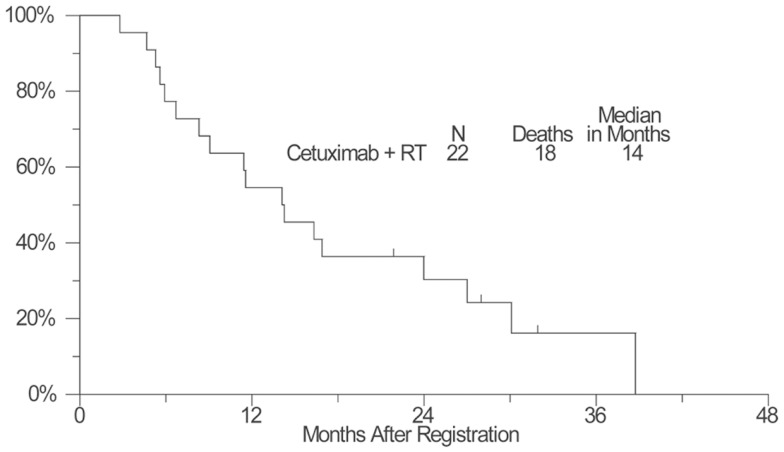
**A plot of Kaplan–Meier estimates of overall survival (OS) of patients treated with concurrent cetuximab and chest radiation**. OS was defined as the time from the date of enrollment until the date of death due to any cause. Patients last known to be alive were censored at the date of last contact and are marked on the curve with a tic representing the last follow-up time.

**Figure 2:**
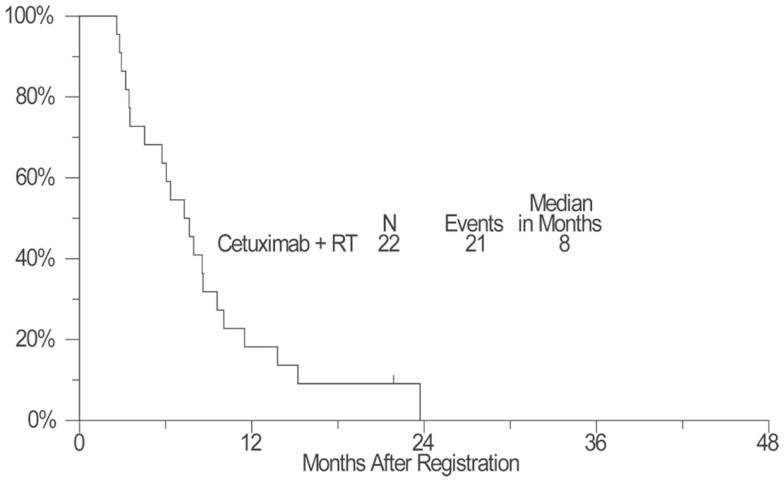
**A plot of Kaplan–Meier estimates of progression-free survival (PFS) of patients treated with concurrent cetuximab and chest radiation**. PFS was defined as the time from the date of enrollment until the date of first documentation of disease progression (per RECIST), symptomatic deterioration, or death due to any cause. Patients last known to be alive and progression-free were censored at the date of last contact and are marked on the curve with a tic representing the last follow-up time.

As of April 11, 2012, four patients remained alive with a median follow-up of 25 months (range: 17–32 months). Kaplan–Meier OS estimate at 1-year survival was 55% (95% confidence interval: 32–72%) and the median OS estimate was 14 months (95% confidence interval: 8–24 months) (Figure 1). The estimated 1-year PFS was 18% (95% confidence interval: 6–36%) and the estimated median PFS was 8 months (95% confidence interval: 5–9 months) (Figure 2).

### EGFR protein expression analysis

As none of the patients in this study received chemotherapy as part of the regimen, this study population represented a unique cohort and opportunity to assess if EGFR expression affected outcome in patients treated with the combination of radiation and cetuximab only. We were aware of the limitation of the small sample size of this study population, thus this is an exploratory analysis. Tumor samples from 11 patients were assayed with IHC using Dako pharmDx. As none of the reported H-scores were at least 200, the data were dichotomized by splitting on H-score ≥100 vs.<100. Analysis of this small cohort of cases did not demonstrate any correlation between Membrane H-score and OS (*p* = 0.76) and PFS (*p* = 0.39), or between Cytoplasmic H-score and OS (*p* = 0.59) and PFS (*p* = 0.87). The tumor histology, membrane, and cytoplasm H-scores vs. PFS, and OS are shown in Table 3.

**Table 3:** **Histology, membrane, and cytoplasm H-scores vs. tumor response, PFS, and OS**.

Case	Histology	Membrane H-score	Cytoplasm H-score	Best response*	PFS (months)	OS (months)	Comment
a	ADENO	150	95	UPR	6.3	38.7	
b	ADENO	100	150	NASS	2.8	2.8	
c	NSCLC, NOS**	65	100	NASS	7.3	16.3	
d	LARGE	40	30	PR	8.6	16.9	Scant/cell block ∼20 cells
e	ADENO	100	75	INC	3.2	5.3	
f	ADENO	60	105	NA	13.8	27.0	
g	NSCLC, NOS	120	55	UPR	9.6	14.3	
h	SQUAM	140	180	PR	23.7	24.0	
i	SQUAM	90	80	STA	21.9	21.9	
j	NSCLC, NOS	20	20	PR	5.7	6.7	
k	SQUAM	15	20	PR	11.5	14.1	
l	SQUAM	10	0	STA	3.5	4.7	

*CR, complete response; UCR, unconfirmed complete response; PR, partial response; UPR, unconfirmed partial response; STA, stable; INC, increasing disease; NASS, assessment inadequate; NA, not applicable; **NOS, not otherwise specified.

## Discussion

While many randomized phase 3 studies have established combination chemoRT treatment as a standard of care for stage III NSCLC patients with good PS, there is no consensus for the treatment of patients with poor-risk features. Outcomes from the two previous SWOG studies, S9429 and S9712, highlight the obstacles of treating poor-risk stage III NSCLC. Balancing therapy intensity with tolerability remains a major challenge. Experience from S9429 and S9712 support the conclusion that concurrent cytotoxic chemotherapy and radiotherapy may not be optimal for the treatment of poor-risk stage III NSCLC patients due to toxicities from therapies.

There are pre-clinical and clinical data supporting the combination of radiation treatment with cetuximab. Pre-clinical studies have demonstrated radiosensitizing effects of cetuximab using lung cancer cell lines *in vitro*, and *in vivo* using human lung cancer xenografts transplanted into mice (19, 20). A phase 3, randomized clinical study for head and neck cancers has demonstrated that cetuximab enhanced radiation treatment effects and improved both local-regional cancer control and survival (11). Both the pre-clinical findings and the clinical study in head and neck cancer lend support to the rationale of combining cetuximab with radiation treatment in enhancing radiation effects.

Our study combining cetuximab and chest radiotherapy did show that concurrent cetuximab and chest radiotherapy was well tolerated. Toxicity assessment revealed 22.7% overall ≥Grade 3 non-hematologic toxicity. Grade 3 esophagitis was observed in one patient (5%). The skin reactions were mostly Grade 1 or 2 except two of 22 (9%) had Grade 3 acne and one of 22 (5%) had Grade 3 radiation skin burn. Grade 3–4 hypomagnesemia was seen in four (18%) patients. One patient (5%) had elevated cardiac troponin and pulmonary emboli. We do caution the interpretation of the observed low rate of Grade 3 esophagitis, since the probability and severity of esophagitis is dependent on esophageal dose and volume, and we do not have information on these parameters. There were no treatment-related deaths. We note no Grade 3 pneumonitis in this study, which may attribute to our stringent normal lung allowance dose of V20 to 30%, compared with most chemoRT studies using 35% as the upper limit. It is also possible that the limitation of V20 to 30% might have selected out some patients with much larger tumor volumes.

The FLEX study randomized 1125 patients with advanced NSCLC to chemotherapy plus cetuximab vs. chemotherapy alone and showed improved OS of chemotherapy plus cetuximab arm (HR 0.87). EGFR protein expression by the H-score assessment was conducted in samples from 1121 patients and showed that high EGFR expression was associated with longer survival in the chemotherapy plus cetuximab group than the chemotherapy alone group (median survival 12.0 vs. 9.6 months, HR = 0.73, *p* < 0.05) with the H-score cut-off value of 200 (15). Surprisingly, in our exploratory study, no patients had a H-score ≥200, which in the FLEX study constituted approximately one third of the patients. When we used an H-score of 100 as cut-off, we did not note a significant difference in survival outcome of patients. The lack of power to discern real differences is expected given the small sample size, unless the association is “highly specific” as was seen in the association between EGFR mutation and the response to EGFR tyrosine kinase inhibitor reported by Lynch et al. (21), when a definite association was detected with a total sample size of 16 (eight of nine responders with EGFR mutation and none of seven non-responders had the mutation).

There are other clinical studies seeking optimal therapy for either the elderly or the poor-risk patients with stage III NSCLC. Atagi et al. (22) reported the outcome of thoracic radiotherapy with or without daily low-dose carboplatin in elderly patients with NSCLC in a randomized, controlled phase 3 trial by the Japan Clinical Oncology Group (JCOG0301). The study allowed for ECOG PS 0–2. Patients older than 70 years with unresectable stage III NSCLC were assigned to chemoRT (60 Gy) plus low-dose carboplatin (30 mg/m^2^/day, 5 days a week for 20 days) or RT alone. They reported a median survival of 22.4 months for the chemoRT arm, and 16.9 months for the RT alone arm. Grade 3–4 hematologic toxic effects were similar between the two groups. The incidences of Grade 3–4 pneumonitis and late lung toxicity were similar between groups as well. There were seven treatment-related deaths: 3 of 100 patients (3%) in the chemoRT group and 4 of 100 (4%) in the RT group. Ready et al. (23) reported a study of two cycles of induction paclitaxel 200 mg/m^2^ and carboplatin AUC 6, plus gefitinib 250 mg daily in patients with stage III NSCLC. Poor-risk stratum 1 (> or = 5% weight loss and/or PS 2) received RT daily 2 Gy for 33 fractions (66 Gy) and gefitinib 250 mg daily. Good-risk stratum 2 (PS 0–1, and weight loss<5%) received the same RT with gefitinib 250 mg daily and weekly paclitaxel 50 mg/m^2^ plus carboplatin AUC 2. Consolidation gefitinib was administered until disease progression. The study yielded a median PFS of 13.4 months and median OS of 19.0 months for poor-risk stratum; and a median PSF of 9.2 months and a median OS of 13 months for good-risk stratum. Survival for good-risk patients receiving concurrent chemoRT plus gefitinib was disappointing even for tumors with active EGFR mutations. This finding highlighted the potential adverse consequences from chemotherapy-related toxicity even in good-risk patients.

To date, there were six phase 2 national and international studies published using cetuximab as part of the treatment for stage III NSCLC (24–29). Five of the six studies were designed for patients with good PS, and one study has allowed both good performance elderly (79%) and younger patients with poor performance (21%) (Table 4). Four of the six studies have combined cetuximab with chemotherapy and radiation treatments, while only two studies combined cetuximab with RT only. Of the two, the study by Jensen et al. (29) applied IMRT to a total dose of 66 Gy in 2 Gy daily fractions in combination with concomitant as well as 13 weekly maintenance cycles of cetuximab. Patients in this study had slightly favorable baseline prognostic factors (inclusion of some stage II patients, no weight loss>5%, and younger median age). This study yielded a median survival of 19 months and a response rate of 63%. The other study by Jatoi et al. (28) applied concurrent cetuximab with 60 Gy in 2 Gy daily fractions of chest RT for patients ages ≥65 years with PS 0–1, or<65 years old with PS of 2. This study yielded a median survival of 17 months, and a 26% response rate. It is clear that all published work of poor-risk patients, elderly patients, and patients treated with combination cetuximab and RT varied in the eligibility criteria of patient selection (Table 4) (24–29). Patient selection bias in phase 1 and 2 studies can confound the interpretation of therapy outcome. Our study was not designed to measure survival as the primary endpoint, and we were fully aware of the limitation of data interpretation from the small sample size in a pilot phase 1 study. Further studies with larger sample sizes will be useful to establish the optimal therapeutic ratio of this regimen. Nevertheless, the median survival of 14 months of our study compared favorably to the two previous SWOG trials for similar eligible patients of poor-risk, stage III population. The slow accrual was primarily due to the individual preferences of therapy regimen by oncologists of SWOG participating institutions in treating poor-risk stage III NSCLC. Defining the optimal therapy for poor-risk stage III NSCLC remains a challenge to oncologists.

**Table 4:** **Clinical studies of combined cetuximab and radiotherapy for stage III NSCLC**.

Clinical Trials	Study phase, sample size	Patient selection (*PS)	Regimen	Survival (month)	Non-hematologic **AE ≥ Grade 3
Swedish Lung Cancer Study Group (26)	Phase 2 *N* = 71	Good PS	Induction docetaxel/cisplatin × 2 then concurrent cetuximab/RT (68/2 Gy)	MS 17 mo. OS 66% at 1-year; 37% at 2-years; 29% at 3-years	1.4% esophagitis; 5.6% hypersensitivity; 15.4% febrile neutropenia; 4.2% skin reaction; 11.3% diarrhea; 4.2% pneumonitis; 1.4% Grade 5 pneumonitis
CALGB 30407 (25)	Randomized phase 2 *N* = 48(arm A) *N* = 53 (arm B)	Good PS	*Arm A*: carbo/pemetrexed × 4, then concurrent ChemoRT (70/2 Gy), then pemetrexed	OS at 18 mo. 58% *Arm A*, vs. 54% *Arm B*	*Arm A*: 52%; *rm B*: 62%; (including esophagitis, dysphagia, fatigue pneumonitis, dehydration, ***N/V) 4% Grade 5 AE-*Arm A*, 5.7% Grade 5 AE-*Arm B*
			*Arm B*: carbo/pemetrexed × 4, then weekly cetuximab and concurrent chemoRT (70/2 Gy), then pemetrexed	
RTOG 0324 (24)	Phase 2 *N* = 87	Good PS	Cetuximab weekly, carbo/paclitaxel/RT 63/1.8 Gy, followed by carbo/paclitaxel × 2 cycles	MS 22.7 mo.; 49.3% OS at 2 years	8% Esophagitis; 7% pneumonitis; 6% Grade 5 AE
The UK SCRATCH Study (27)	Phase 1 *N* = 12	Good PS	Platinum-based induction chemo, followed by concurrent weekly cetuximab/RT 64/2 Gy	OS 66.7% at 1 year	8.3% Pneumonitis; 8.3% lethargy
NCCTG Study N0422 (28)	Phase 2 *N* = 57	Elderly ≥65 years/old and good PS; and/or<65 years/old and poor PS	Concurrent weekly cetuximab/RT, 60/2 Gy	MS 15.1 mo.; Median PFS 7.2 mo.	40% Overall AE, including fatigue, anorexia, dyspnea, rash, dysphagia
The German NEAR Trial (29)	Phase 2 *N* = 30	Good PS	Concurrent cetuximab/^#^IMRT, 66/2 Gy, followed by 13 weekly maintenance cetuximab	MS 19.5 mo.; OS 66.7% at 1-year; 34.9% at 2-years; Median PFS 8.5 mo.	36.7% Overall AE; 3.3% Grade 3 pneumonitis
SWOG 0429 (current study)	Phase 1 pilot study *N* = 22	Poor PS, or good PS with poor PFT and co-morbidities	Concurrent cetuximab/RT (64.8/1.8 Gy) followed by weekly cetuximab till 2-years or disease progression	MS 14 mo.; OS 55% at 1-year; Median PFS 8 mo.; PFS 18% at 1-year	22.7% Overall AE, including 18% hypomagnesemia; 9% acne; 5% skin burn; 5% esophagitis; 5% Grade 4 cardiac troponin/^##^PE

*PS. performance status.

**AE, adverse events.

***N/V, nausea/vomiting.

*^#^IMRT, intensity modulated radiotherapy*.

*^##^PE, pulmonary emboli*.

## Conflict of Interest Statement

The authors declare that the research was conducted in the absence of any commercial or financial relationships that could be construed as a potential conflict of interest.
